# New Work on the Climate of the United States, and Its Endemic Diseases

**Published:** 1841-12

**Authors:** 


### NEW WORK ON THE "CLIMATE OF THE UNITED STATES AND ITS ENDEMIC INFLUENCES."

Dr. Samuel Forry, of the Medical Staff of our army, whose "*Statistical Researches relative to the Ætiology of Pulmonary and Rheumatic diseases,*" were noticed in a late number of this journal, has issued proposals for a new work under the above title. It will be based on the army registers and returns, but in rearing the superstructure the author will draw materials from other sources. Judging from what we have seen of his labours, we cannot doubt that his undertaking will be ably executed; and that he will, to a great extent, supply a desideratum which all must admit to exist. The work will extend to 350 pages. Price, two dollars and fifty cents. Such of our readers as have intercourse with Louisville, may subscribe for it at the Library of the Institute and have their copies sent to this city. D.

